# Permanent Cardiac Pacing of the Right Ventricular Outflow Tract Guided by Real-time Assessment of Electromechanical Synchrony

**DOI:** 10.19102/icrm.2024.15042

**Published:** 2024-04-15

**Authors:** Alejandro Ventura, Luciana Viola, Andrés Di Leoni Ferrari

**Affiliations:** 1 Cordis Instituto del Corazón, Resistência, Argentina; 2 Cardiac Pacing Unit, Cardiology Service, Hospital São Lucas da PUCRS, Porto Alegre, Brazil

**Keywords:** Apical pacing, artificial cardiac pacing, cardiomyopathy, permanent pacemaker

## Abstract

Permanent right ventricular apical pacing deteriorates cardiac systolic function in some patients. We investigated an alternative site for permanent pacemaker (PPM) lead positioning with the goal of achieving more physiological pacing. A total of 132 patients with bradyarrhythmias underwent PPM implantation at the right ventricular outflow tract (RVOT) with conventional active-fixation leads. A real-time cross-correlation analysis (CCA) was performed using the Synchromax^®^ software (EXO Health, Seattle, WA, USA) to determine the optimal site for ventricular lead implantation based on the cardiac synchrony index. The follow-up period ranged from 6–36 months, and the following parameters were assessed: pacing capture threshold, lead stability, and the need for lead repositioning. Adequate parameters were achieved in 129 patients (98%), and there were no procedure-related complications. At follow-up, no leads were dislodged, pacing thresholds remained stable, and no lead required repositioning. Using real-time CCA as an intraoperative parameter during PPM implantation at the septal RVOT helps to achieve cardiac synchrony in the vast majority of cases. This technique is a simple, effective, and safe way to simplify and standardize PPM implantation at the RVOT.

## Introduction

In artificial cardiac pacing (ACP), the ventricular lead is regularly implanted at the right ventricular (RV) apex (classically named “conventional pacing”) due to the ease of placement, lead stability, and the safety of the surgical procedure.^1^ However, conventional RV apical pacing produces an activation sequence similar to that of left bundle branch block (LBBB), characterized by abnormally late depolarization of the left ventricular (LV) lateral wall and electromechanical asynchrony, with serious clinical consequences.^1,2^ Changes in hemodynamics and mechanical activity resulting from this abnormal pattern of activation cause ventricular remodeling related to LV cell anomalies, both at the macroscopic and ultrastructural levels, with electrophysiological and neurohormonal consequences.^3–6^ In ACP-dependent patients with a high RV pacing burden, clinical consequences include an increased risk of developing systolic LV dysfunction, heart failure, and atrial fibrillation, among others.^3,6,7^ These adverse effects prompted the search for alternative RV pacing sites (outside the apex) with the aim of achieving a more physiological activation sequence.^8^ Such sites include the conduction system (His-bundle pacing and deep septal pacing of the left bundle branch area), para-Hisian region, and RV outflow tract (RVOT).

Conduction system pacing (CSP) has emerged as the most physiological pacing approach to correct electrical dyssynchrony. Despite its effectiveness, many technical and clinical challenges persist, including difficulties in delivering the lead, ensuring accurate capture confirmation, maintaining stable pacing thresholds over time, and ensuring long-term lead stability.^9^

Consensus has not been reached on the use of either non-selective or selective His-bundle pacing, and the widespread use of these techniques is precluded by their complexity, costly equipment, increased risk of complications and reinterventions, high pacing thresholds, shorter battery life, uncertainty regarding long-term safety, and a significantly longer learning curve.^4,10^ In addition, a mapping system and the presence of an electrophysiologist trained on His-bundle mapping are often required during these procedures, which may constitute another limitation, as cardiac pacing devices are usually implanted by cardiovascular surgeons in developing countries. Conversely, increasing evidence has led to the rapid adoption of CSP via left bundle branch area pacing (LBBAP), which is more straightforward and capable of overcoming the downside of His-bundle pacing.^9,11^ However, it requires the implantation of more complex pacing systems and the use of dedicated equipment.

Placing the lead in the RVOT is less complex than pure CSP and may be performed without the use of special equipment. RVOT pacing showed initial favorable outcomes compared to RV apical pacing.^12–14^ ACP parameters (under bipolar configuration: R-wave detection, pacing threshold, and impedance) are usually adequate, and reintervention due to lead instability is rarely required.^15^ The technique commonly used to pace the septum and the RVOT, based on fluoroscopy and electrocardiographic (ECG) patterns, showed heterogeneous results, indicating that the reference parameters alone are not sufficient to objectively determine the optimal implant site.^4,12,16,17^ These barriers could be overcome with the use of the cross-correlation analysis (CCA) carried out by Synchromax^®^ (EXO Health, Seattle, WA, USA) to determine the optimal pacing site within the RVOT. CCA is a non-invasive, sensitive, and practical method with a high predictive value for detecting LV electrical synchrony and asynchrony and was also shown to have a very good correlation with synchronous mechanical activity.^8,10,18–20^ The use of CCA during permanent pacemaker (PPM) implantation at the RVOT can simplify and standardize the implant procedure, making synchronous electromechanical activity more accessible and achievable. The objective of this single-center, retrospective study was to investigate an alternative site (RVOT) for PPM lead implantation with the goal of achieving more physiological pacing.

## Materials and methods

**Figure 1:**
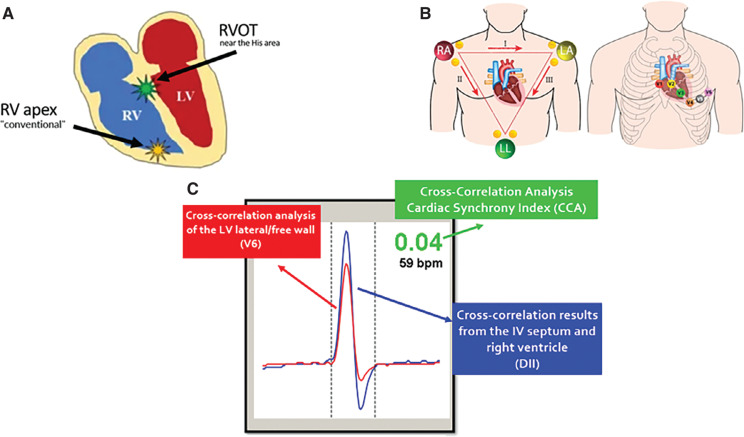
Permanent right ventricular outflow tract pacing guided by real-time assessment of electromechanical synchrony. **A:** Drawing of the heart showing the different locations of the pacing leads and the proximity of the right ventricular outflow tract in relation to the His area when compared with the right ventricular apex. **B:** Electrocardiogram leads from which the cross-correlation analysis is generated; DII corresponds to the septum and right ventricle, while V5–V6 corresponds to the left ventricle. **C:** Screen displaying cross-correlation curves and the cross-correlation analysis. *Abbreviations:* IV, interventricular; LA, left arm; LL, left leg; LV, left ventricle; RA, right arm; RV, right ventricle; RVOT, right ventricular outflow tract.

**Figure 2:**
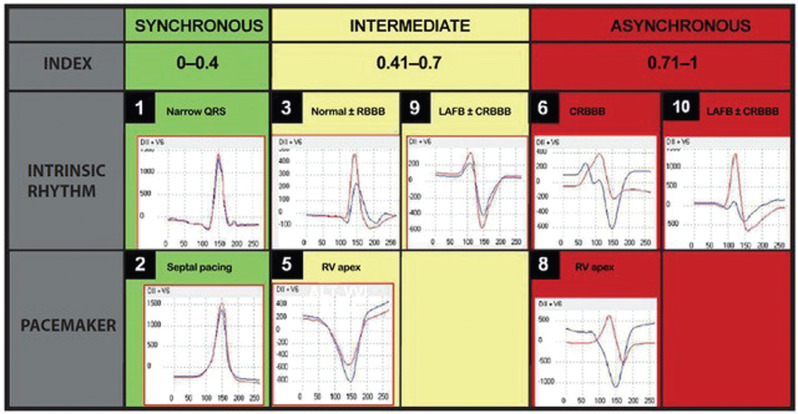
Synchromax^®^ curves showing different QRS morphologies on electrocardiograms of intrinsic rhythm (upper panel) and artificial cardiac pacing (lower panel) with a narrow QRS or conduction abnormalities. Normal/right bundle branch block/left anterior fascicular block/left bundle branch block according to cardiac synchrony index values and pacing site (septal/right ventricular apex). Blue lines: lead II (septum and right ventricle); red lines: lead V6 (left ventricle). *Abbreviations:* CRBBB, complete right bundle branch block; LAFB, left anterior fascicular block; RBBB, right bundle branch block; RV, right ventricular.

From April 2019 to April 2023, 132 patients underwent PPM implantation at the RVOT. Fifty-six were women (43%), with a mean age of 62 years (standard deviation [SD], 9.6 years). Thirty-one devices were single-chamber devices and 101 were dual-chamber devices. Indications for PPM implantation included sinus node disease in 39 patients, complete atrioventricular block (AVB) in 66, second-degree AVB in 19, and complete LBBB or bifascicular block in 8, respectively. As for the underlying cardiomyopathy, 54 patients had hypertension/sclerodegenerative lesions, 15 had Chagas disease, 36 had ischemic necrosis, 21 had dilated cardiomyopathy, and 6 had heart valve disease **(Table 1)**. Conventional active-fixation leads were used in all implants. CCA was performed in real time during the procedure to determine the optimal place for lead implantation at the RVOT according to the cardiac synchrony index (CSI) values **(Figure 1)**. The CSI is a mathematical index generated by Synchromax^®^, in which a value of 0.00–0.40 corresponds to synchrony, that of 0.40–0.70 corresponds to poor synchrony, and that of >0.71 corresponds to dyssynchrony **(Figure 2)**.

Synchromax^®^ was designed to identify electrical dyssynchrony non-invasively by spectral and averaging analyses of many QRS complexes from leads DII and V6, which correspond to septum and LV lateral wall activation, respectively.^8,19,20^ This analysis generates a blue (DII) and a red (V6) curve, which inform the direction of activation (from the base to the apex or vice versa), the simultaneity of activation, and impulse propagation delay (QRS width).

The study was conducted in accordance with the Declaration of Helsinki and was approved by the ethics committee of our institution. The requirement for informed consent was waived due to the retrospective nature of the study.

### Implant technique

**Figure 3:**
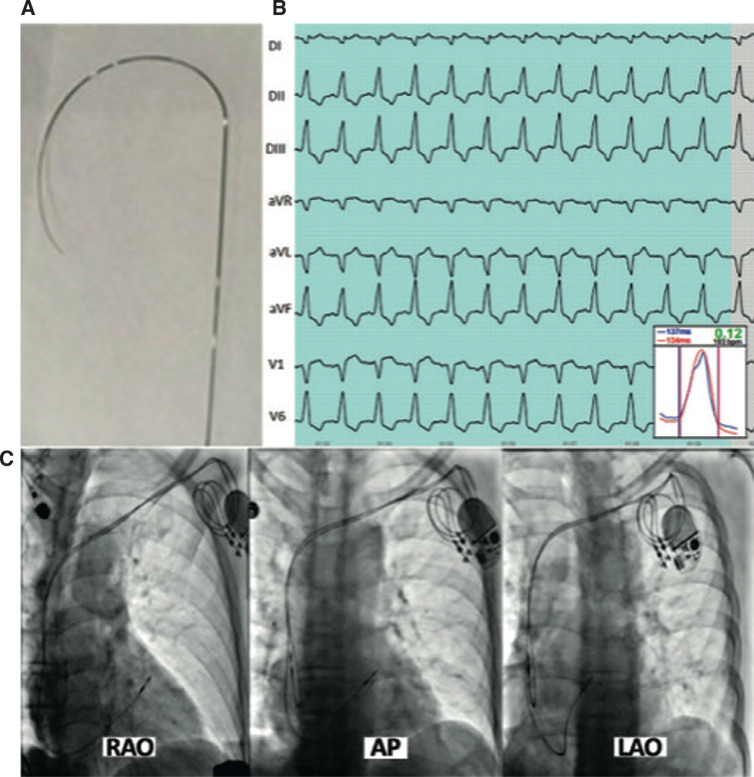
**A:** Ventricular lead stylet with a curvature on the distal end. **B:** Electrocardiogram pattern and Synchromax^®^ curves, with a cardiac synchrony index value of 0.12. **C:** Fluoroscopy of the same patient in the right anterior oblique, anteroposterior, and left anterior oblique views showing the permanent pacemaker lead implanted at the septal right atrium (para-Hisian). *Abbreviations:* AP, anteroposterior; LAO, left anterior oblique; RAO, right anterior oblique.

According to a previously described method,^8^ a conventional active-fixation bipolar lead was inserted into the right atrium. The stylet was removed, and two curves were performed on its distal end **(Figure 3A)**. The lead was then mounted on the stylet and, during posteroanterior fluoroscopy, advanced into the pulmonary artery and gently pushed downward until it reached the RVOT, below the pulmonary valve. Next, in the left anterior oblique (LAO) view (30°), the stylet was retracted 3–4 cm in order to free the lead tip and allow support over the septum and RVOT, with the latter being directed toward the patient’s spine in this view. After delivering an output voltage of 5 V at 10 bpm higher than the patient’s native heart rate, the RVOT was mapped with the goal of achieving 100% capture as well as curves and CSI values suggestive of cardiac synchrony (CSI ≤ 0.40) **(Figure 3B)**. Once this was achieved, the lead was permanently fixed, and the following electronic parameters of interest were measured: pacing threshold, impedance, and R-wave amplitude (detection). If any of these parameters were suboptimal, mapping was performed again. For a mean follow-up period of 6–36 months, pacing threshold, lead stability, and the need for lead repositioning were assessed every 6 months.

Statistical analysis was performed using SPSS Statistics for Windows version 27.0 (IBM Corporation, Armonk, NY, USA). Categorical variables are expressed as proportions (%), and quantitative variables are presented as measures of central tendency (mean) and dispersion (SD).

## Results

Of the 132 patients who underwent PPM implantation at the RVOT, synchrony curves and values with adequate implant parameters were achieved in 129 (98%). The mean CSI was 0.20 ± 0.11 (0.07–0.38), and the mean bipolar pacing threshold was 0.8 ± 0.7 V (0.4–1.1 V) **(Table 2)**. In three patients without native heart rhythms, the lead had to be placed at the RV apex due to lead dislodgement or instability during the procedure, which precluded mapping of the septal RVOT. There were no other procedure-related complications. At follow-up, no leads were dislodged, and electronic parameters remained stable.

## Discussion

In this study, an alternative approach to placement of active-fixation leads in the RVOT with the aim of achieving a more physiological pacing response is presented, compared to traditional fluoroscopic and ECG-only approaches. We were able to demonstrate the safety and efficacy of achieving cardiac synchrony using a simple and inexpensive CCA-guided pacing technique in 98% of cases. Standardization of PPM implantation at the RVOT using real-time CCA may be a starting point for the future development of new ACP strategies with clinical results that are not inferior to CSP, particularly non-selective His-bundle pacing.^8^

The great usefulness of CCA-guided lead placement at the RVOT is that it is a simple procedure that does not involve extensive operating time, specific materials, or specific training. Achieving physiological pacing should always be the goal when implanting a pacing device, even in patients with a normal heart and narrow QRS complex, as it is important to avoid any damage that could be caused in patients without dyssynchrony. Cross-correlation is a measure of similarity between two signals as a function of the displacement of one relative to the other. Intrinsic conduction and simultaneous QRS complexes from different leads tend to minimize the phase between them—that is, the R peaks tend to align spatially. However, when conduction pathways are impaired, leads projecting onto fibrotic or non-conducting myocardium show delayed spikes as well as changes in morphology that affect their similarity to healthy pathways. This is the basis for the CCA used in this study.^18^

The commonly used technique for permanent septal RVOT pacing, based on fluoroscopy and ECG patterns, has not yielded adequate or sufficient results to standardize the technique, failing to objectively determine the optimal pacing site.^4,12,13,16,17,21^ The anatomical complexity associated with the RVOT may explain some of these difficulties.

The term RVOT in the setting of ACP has been used to describe a variety of anatomical sites, including the true RVOT, mid-septum, and para-Hisian region (surrounding the His bundle), among others. Despite efforts to standardize the nomenclature of nonapical ACP sites, this confusion persists.^22,23^

The RVOT composes the septum posteriorly and the free wall anteriorly, and, between them, there is a narrow border where the left anterior descending coronary artery is located, in addition to a thicker posterior border.^1,22^ The RVOT is bounded by the pulmonary valve superiorly and the superior aspect of the tricuspid apparatus and supraventricular crest inferiorly, with the latter extending into the RV free wall with the septomarginal trabecula and moderator band.^24^ The inferior margin can be considered as a line drawn from the superior tricuspid annulus (in whose superior end the His bundle is located) to the interventricular septum.^22,25^

The anatomical lead position at the RVOT according to different radiographic views is shown in **Figure 3C**.^4,22,24^ Previous studies focused on implanting the lead in the septal RVOT so that, once the lead was positioned in the RVOT on the anteroposterior and right anterior oblique views, the septal position of the lead was automatically confirmed on the LAO view, in which the tip of the lead is directed toward the left (the patient’s spine) **(Figure 3)**.^1,4,5,25^

### Correlations between electrocardiographic morphology and lead position

Favorable studies have shown that lead implantation at the septal RVOT is an alternative physiological pacing site because of its proximity to the conduction system in a region closer to the Purkinje system (His-bundle area pacing—a concept that deserves further investigation).^26,27^ This type of pacing would produce synchronous interventricular contraction characterized by a narrower QRS complex and could prevent the deterioration of LV structure and function. However, clinical evidence supporting this strategy is still scarce and unclear. Although some studies have reported that RV septal pacing could reduce LV dyssynchrony, a retrospective control study^28^ and a prospective randomized controlled trial^16^ failed to demonstrate the prognostic benefit of RV septal pacing, partially due to the low success rate of RV lead placement at the RV septum.

**Figure 4:**
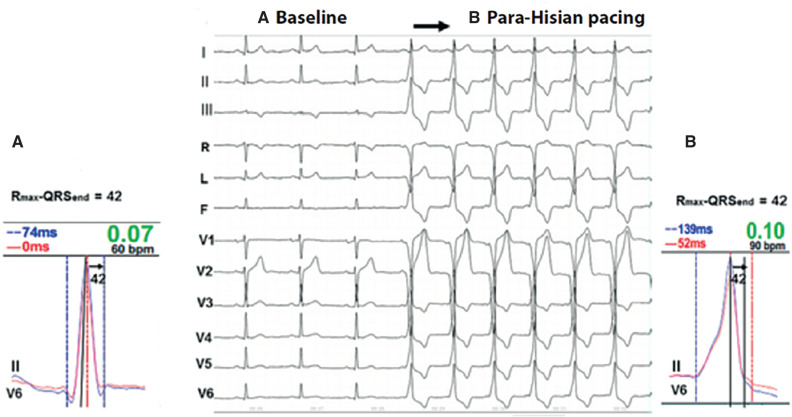
Electrocardiogram of **(A)** sinus rhythm versus **(B)** artificial cardiac pacing at the right ventricular outflow tract. Synchromax^®^ showing identical baseline and paced R_max_–QRS_end_ intervals, indicating that the stimulus penetrates and activates the conduction system with a delay. Reused with permission from de Zuloaga C, Ferrari ADL. Electrophysiological demonstration of nonselective His-Purkinje system capture with para-Hisian pacing. *J Electrocardiol*. 2023;79:38–45.

ECG patterns are of the utmost importance to determine the lead position in the RVOT. It should be noted that the septal RVOT is located more posteriorly than the free wall, which is reflected on typical paced ECG patterns. Septal ACP typically produces narrower QRS complexes than the free wall, as well as a negative or isoelectric vector on DI.^22^ Thus, this feature has a positive predictive value of 90% for septal placement. Conversely, free-wall ACP is associated with a wide QRS interval, notching in inferior leads (DIII), and a positive vector in lead DI **(Figure 4B)**.^29^

#### Right ventricular outflow tract pacing with cardiac synchrony despite a slightly wider QRS

Although many studies are still ongoing, the concept of CSP has been widely adopted, and its benefits are recognized as a promising ACP strategy. There is growing evidence that CSP by either selective or non-selective His-bundle capture, or the new perspective offered by LBBAP^9,11^ (although requiring implantation of more complex systems with dedicated equipment), is markedly superior to other pacing sites because it produces a physiological sequence of cardiac activation and contraction.^1^

However, according to Brignole and Sutton,^30^ the strongest rationale supporting the benefit of CSP is shortening of the QRS duration. However, as observed in the MELOS registry,^31^ CSP and, therefore, the expected physiological sequence of electromechanical activation and contraction are associated with a wider QRS. Thus, although CSP can achieve electrical LV synchrony in some patients, the observed average wide QRS during physiological pacing suggests that, in most patients, particularly those with advanced heart failure (candidates for biventricular pacing), delayed activation of the LV lateral wall might result from distal conduction system delay, electrical uncoupling, myocardial scars, or functional conduction blocks.^30^

Conversely, the use of CCA showed that RVOT pacing generates inter- and intraventricular synchrony despite producing a wider QRS complex. Our results are similar to those of previous studies,^8,19,29^ in which analysis of variance of the QRS (Synchromax^®^)—referred to as CCA in this study—showed higher sensitivity and a negative predictive value for detecting mechanical dyssynchrony when compared to QRS duration on conventional ECG. Furthermore, these studies found no correlation between QRS duration and mechanical dispersion based on speckle-tracking strain of the left ventricle, confirming that mechanical dyssynchrony evidenced by echocardiography (ECHO) cannot be fully understood when strictly based on standard ECG.

**Figure 5:**
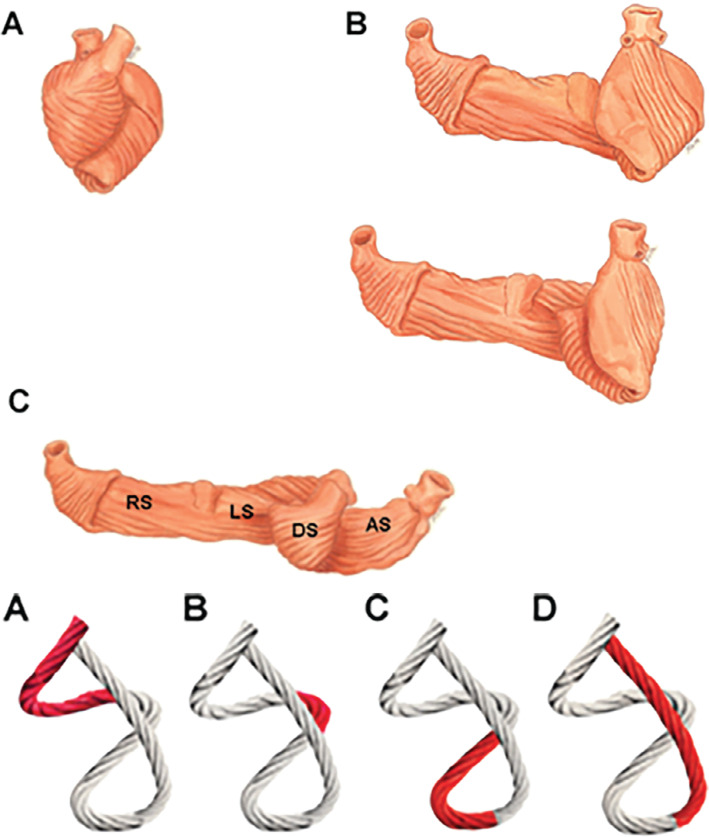
Helical ventricular myocardial band of Torrent-Guasp. Upper panel: **A, B:** Unrolling of the ventricular myocardium into a single muscle band. **C:** Extended muscle band showing the right and left segments and the ascendant and descendant segments. Lower panel: Rope model of the muscle band showing the sequence of electrical activation (red) in the different sections. **(A)** Ventricular activation initiates at the right basal segment (right ventricular outflow tract) and travels to the left basal segment, **(B)** subsequently descending in an oblique fashion from the lateral aspect of the left ventricle toward the septum **(C)** and taking a turn at the tip. **D:** Finally, it runs vertically upward through the ascending segment that ends in the aorta, causing mechanical ventricular ejection.

Possible explanations for this phenomenon include the following:

- Interval between peak R-wave and the final segment of the QRS complex (R_max_–QRS_end_): the His–Purkinje system begins at the crest of the interventricular septum, immediately below the membranous septum, and is subsequently divided into the left and right branches, which run along the medial septum until they reach the Purkinje fibers. It was recently demonstrated by de Zuloaga and Ferrari^29^ that RVOT pacing (such as non-selective His-bundle capture) produces a paced QRS complex that is slightly wider than the baseline one. However, the R_max_–QRS_end_ interval remains the same, which indicates the presence of inter- and intraventricular cardiac synchrony, confirming that, after rapidly passing through non-specific cardiac muscle, the stimulus penetrates the His–Purkinje and captures it. Therefore, ventricular activation and contraction are mainly mediated by the specific conduction system. Muscle activation produced by septal RVOT pacing is represented by a pseudo-delta wave on the ECG **(Figure 4)**.^29^
- Helical ventricular myocardial band (HVMB): in the middle of the last century, Torrent-Guasp described new observations on the anatomy and function of the myocardium based on the dissection of animal and human hearts.^32,33^ The HVMB concept of Torrent-Guasp **(Figure 5)**^34^ was revolutionary by changing the deeply rooted concept in the scientific community that the cardiac muscle functions as a syncytium of disorganized and intertwined cells that contract radially in systole. In summary, it suggests that the myocardium is in fact a single muscle band that twists on itself. The muscle band begins at the RVOT and is then divided into two loops: the basal and the apical loop. The basal loop, in turn, is divided into two segments: the right segment, coinciding with the RV free wall, and the left segment, coinciding with the left ventricle. The apical loop is next, which is also divided into two segments. The descending segment includes muscle fibers that descend diagonally toward the septum and then make a turn around the apex, from where the fibers ascend almost vertically toward the base of the heart (ascending segment), ending at the aorta **(Figure 5)**.^35,36^ This anatomical arrangement promotes LV systolic torsion, in which the apex and base rotate in opposite directions—while the heart’s apex is fixed, the base is pulled down by a piston-like mechanism, shortening the longitudinal diameter of the left ventricle, which facilitates ventricular blood ejection toward the aorta.^37^ This theory also suggests that early diastole is an active mechanism of contraction rather than relaxation, in which ascendant muscle fibers elongate the longitudinal diameter of the base/apex when contracting, with closed valves and, consequently, a suction effect due to negative pressure.^38^

These concepts were further developed by the Argentine cardiovascular surgeon and researcher Trainini.^39,40^ In addition to reaffirming, completing, and complementing Torrent-Guasp’s findings, Trainini also described the cardiac fulcrum, which consists of a bony or cartilaginous structure found at the base of the heart between the aortic and pulmonary valves. This structure, which is also found in animals, would work as a support axis so that systolic muscle contraction is effective and has the capacity to generate enough force to eject blood with the necessary speed and pressure.

These theories are closely related to the subject discussed in this article, as both investigators found that all mechanical activity related to cardiac systole arises from the RVOT. The depolarization front would then move toward the apex, preferentially following the longitudinal direction of the myocardial fibers (anisotropy), synchronically activating the septal and lateral surfaces of the left ventricle (**Figure 5**, lower panel) and thereby corroborating the assumption that septal RVOT pacing generates ventricular synchrony despite the presence of a wide QRS complex.

This study has some limitations, particularly the small sample size, single-center design, and lack of a control group undergoing pacing at other RV sites. In addition, we did not record the total number of attempts to locate the final optimal site for RVOT pacing using the CSI. Other limitations include the fact that our analysis was limited to the electrical aspect of ventricular activation and, therefore, to the assessment of cardiac synchrony. As previously discussed, we did not account for mechanical synchrony (ECHO) and pre- and postimplantation hemodynamic LV characteristics, and we did not compare the clinical conditions of the patient population. Finally, this study could have benefited from an intrapatient comparison between RV apical pacing and RVOT pacing using the CCA and a complete follow-up assessment of cardiac dimensions and LV ejection fraction using ECHO, but neither was performed due to methodological and geographical reasons. A scientifically rigorous multicenter study should be conducted in the future to address these aspects.

## Conclusions

Using real-time CCA as an intraoperative parameter during PPM implantation at the septal RVOT helps to achieve cardiac synchrony in the vast majority of cases. This technique is a simple, effective, and safe way to simplify and standardize PPM implantation at the RVOT. The R_max_–QRS_end_ and HVMB hypotheses are not opposite; in fact, they could be complementary and could explain the physiological sequence of cardiac activation and contraction obtained despite the width of the QRS.

## Figures and Tables

**Table 1:** Baseline Patient Characteristics

Characteristics	
Age in years, mean (SD)	62 (9.6)
Female sex	56 (43%)
Underlying cardiomyopathy	
Atherosclerotic disease/hypertension	54 (40.9%)
Ischemia	46 (34.8%)
Chagas disease	15 (11.3%)
Dilated cardiomyopathy	11 (8.3%)
Heart valve disease	6 (4.5%)
Left ventricular ejection fraction, mean (SD)	47.3% (5.3%)
Indications for PPM	
CAVB	66 (50%)
Second-degree AVB	19 (14.3%)
Sinus node disease	39 (29.5%)
Fascicular block	8 (6%)
Type of PPM	
Single chamber (VVI)	31 (23%)
Dual chamber (DDD)	101 (77%)

*Abbreviations:* AVB, atrioventricular block; CAVB, complete atrioventricular block; PPM, permanent pacemaker; SD, standard deviation. Data are presented as n (%), unless otherwise specified.

**Table 2:** Pacing Parameters After Artificial Cardiac Pacing

Parameter	Result
RV outflow tract implantation	129 (98%)
CSI values	0.20 ± 0.11
R-wave detection (mV)	3.2 ± 5
Impedance (Ω)	536 ± 90
RV pacing threshold (V)	0.8 ± 0.7 (bipolar configuration)
Fluoroscopy time (min)	7 (5.2–21)
Paced QRS duration (ms)	132 ± 5

*Abbreviations:* ACP, artificial cardiac pacing; CSI, cardiac synchrony index; RV, right ventricular.
